# Impact of needle-based confocal laser endomicroscopy on the therapeutic management of single pancreatic cystic lesions

**DOI:** 10.1007/s00464-019-07062-9

**Published:** 2019-08-13

**Authors:** Maxime Palazzo, Alain Sauvanet, Rodica Gincul, Ivan Borbath, Goeffroy Vanbiervliet, Raphaël Bourdariat, Anne-Isabelle Lemaistre, Bertrand Pujol, Fabrice Caillol, Laurent Palazzo, Alain Aubert, Frédérique Maire, Louis Buscail, Marc Giovannini, Sébastien Marque, Bertrand Napoléon

**Affiliations:** 1 Hôpital Beaujon, Clichy, France; 2 Hôpital Privé Jean Mermoz, Ramsay Générale de Santé, 4 rue Jacqueline Auriol, 69008 Lyon, France; 3 Cliniques Universitaires, Saint-Luc, Belgium; 4 Centre Hospitalier Universitaire de l’Archet 2, Nice, France; 5 Biomnis, Lyon, France; 6 Institut Paoli Calmettes, Marseille, France; 7 Clinique du Trocadéro, Paris, France; 8 Hôpital Rangueil, Toulouse, France; 9 Capionis, Bordeaux, France

**Keywords:** EUS-FNA, Pancreatic cysts, Needle-based confocal laser endomicroscopy

## Abstract

**Background and aim:**

The diagnosis and therapeutic management of large single pancreatic cystic lesions (PCLs) represent major issues for clinicians and essentially rely on endoscopic ultrasound fine-needle aspiration (EUS-FNA) findings. Needle-based confocal laser endomicroscopy (nCLE) has high diagnostic performance for PCLs. This study aimed to evaluate the impact of nCLE on the therapeutic management of patients with single PCLs.

**Methods:**

Retrospective and comparative study. Five independent pancreatic disease experts from tertiary hospitals independently reviewed data from a prospective database of 206 patients with single PCL, larger than 2 cm and who underwent EUS-FNA and nCLE. Two evaluations were performed. The first one included the sequential review of clinical information, EUS report and FNA results. The second one included the same data + nCLE report. Participants had to propose a therapeutic management for each case.

**Results:**

The addition of nCLE to EUS-FNA led to significant changes in therapeutic management for 28% of the patients (*p* < 0.001). nCLE significantly increased the interobserver agreement of 0.28 (*p* < 0.0001), from 0.36 (CI 95% 0.33–0.49) to 0.64 (CI 95% 0.61–0.67). nCLE improved the rates of full agreement among the five experts of 24% (*p* < 0.0001), from 30 to 54%. With nCLE, the surveillance rate of benign SCAs fell by 35%, from 40 (28/70) to 5% (4/76).

**Conclusion:**

The addition of nCLE to EUS-FNA significantly improves reliability of PCL diagnosis and could impact the therapeutic management of patients with single PCLs. ClinicalTrials.gov number, NCT01563133.

**Electronic supplementary material:**

The online version of this article (10.1007/s00464-019-07062-9) contains supplementary material, which is available to authorized users.

The therapeutic management of pancreatic cystic lesions (PCL) depends on the cyst type. Surveillance or surgical resection is considered for premalignant lesions [mucinous cystic neoplasm (MCN), branch-duct intraductal papillary mucinous neoplasm (BD-IPMN), and neuroendocrine neoplasm (NEN)], while the absence of any surveillance is usually proposed for benign cysts [serous cystadenoma (SCA), pseudocyst (PC)] [1]. When the diagnosis of a single PCL larger than 2 cm remains uncertain after conventional imaging, endoscopic ultrasound fine-needle aspiration (EUS-FNA) with cyst fluid analysis is proposed. Despite the quality of the information provided by EUS, it is not possible to establish the nature of cysts in up to 30% of cases [2]. FNA cytohistology is inconclusive in more than 50% of the cases [3], and the carcinoembryonic antigen (CEA) level lacks specificity [4]. The overall diagnostic accuracy remains low at 61% for both EUS and EUS-FNA [2]. The selection of the most appropriate treatment regimen relies on an accurate characterization of cysts and, therefore, remains suboptimal [5–8]. The consequences of inappropriate treatment for patients in terms of mortality and morbidity are non-negligible with the current standard of care [9–11].

Needle-based confocal laser endomicroscopy (nCLE) is a new imaging modality allowing in vivo real-time imaging at a microscopic level of the inner wall of pancreatic cyst during an EUS-FNA procedure. nCLE feasibility and safety for the assessment of PCLs were demonstrated in a pilot study [12] and were validated in an international multicenter study [13]. Subsequent clinical investigations described the correlation between nCLE images and histological features, and comprehensive nCLE criteria were established for the characterization of the most frequent types of PCL (NEN, MCN, BD-IPMN, SCA, and PC) [14–16]. A recent international study confirmed excellent interobserver and intraobserver agreement for the interpretation of nCLE images for the diagnosis of PCLs [17]. The CONTACT2 clinical trial prospectively validated the very high nCLE sensitivity and specificity for the diagnosis of PCL on a large multicentric series of 78 cases with definitive diagnoses (surgery or cytohistology) [18]. It demonstrated that nCLE diagnostic performance significantly surpassed that of EUS and CEA titration for differentiating mucinous from non-mucinous lesions and benign from premalignant PCLs [18]. Nevertheless, no study has yet evaluated the impact of nCLE on therapeutic management in clinical practice in patients with PCLs. This second phase of the CONTACT2 study aimed to evaluate nCLE impact on therapeutic management through a retrospective analysis of the 206 prospectively included patients with a single PCL.

## Materials and methods

### Study population

The population has been described in the first phase of the CONTACT2 trial published by Napoleon et al. [18]. The inclusion criteria were as follows: diagnosis of large (≥ 20 mm) single PCL identified with CT and/or MRI with at least one cavity larger than 13 mm (to allow for CEA and cytohistopathological analyses) without evidence of communication with the main pancreatic duct, without chronic calcifying pancreatitis; no evidence of criteria for malignancy (cyst containing solid masses or mural nodules, presence of metastatic nodes, presence of distant metastases, ascites and vascular infiltration); and scheduled for a diagnostic EUS-FNA procedure. The CONTACT study protocol was approved by the Institutional Review Board of the Institut Paoli Calmettes (Marseille, France), by the French Health Authority (Agence Française de Sécurité Sanitaire des Produits de Santé) and was registered on ClinicalTrials.gov with the following identifier: NCT01563133. The study was performed in accordance with Good Clinical Practice guidelines. All the co-authors had access to the study data and reviewed and approved the final manuscript.

### Consensus on therapeutic management

Five pancreatic disease experts (three endosonographers with EUS-FNA expertise and two pancreato-biliary surgeons) from four tertiary hospitals were involved. They were independent from the CONTACT2 prospective study. A consensus to standardize the therapeutic management of patients with PCLs according to the diagnosis and its confidence level was first defined by the panel of experts as follows:

1. Eight diagnostic options were retained: SCA, MCN, BD-IPMN, PC, NEN, indeterminate mucinous lesion, indeterminate lesion, and other cyst types (including cystic solid pseudopapillary neoplasm, congenital pancreatic cyst, and cystic lymphoma).
2. Three therapeutic management options were retained: “neither surgery nor surveillance”, “surveillance”, or “surgery”. If the diagnosis was certain, “neither surgery nor surveillance” was recommended for SCAs and PCs; surgery was recommended for MCNs and NENs; either surgery or surveillance was recommended for BD-IPMNs depending on worrisome features observed in EUS; and either surgery or surveillance was recommended for indeterminate mucinous lesions and for indeterminate lesions. The therapeutic management of other rare types of cysts relied on cyst aetiology. Confidence level modified therapeutic management. For example, surveillance instead of “neither surveillance nor surgery” was recommended for SCAs associated with fair confidence level.

### Expert training

**Fig. 1:**
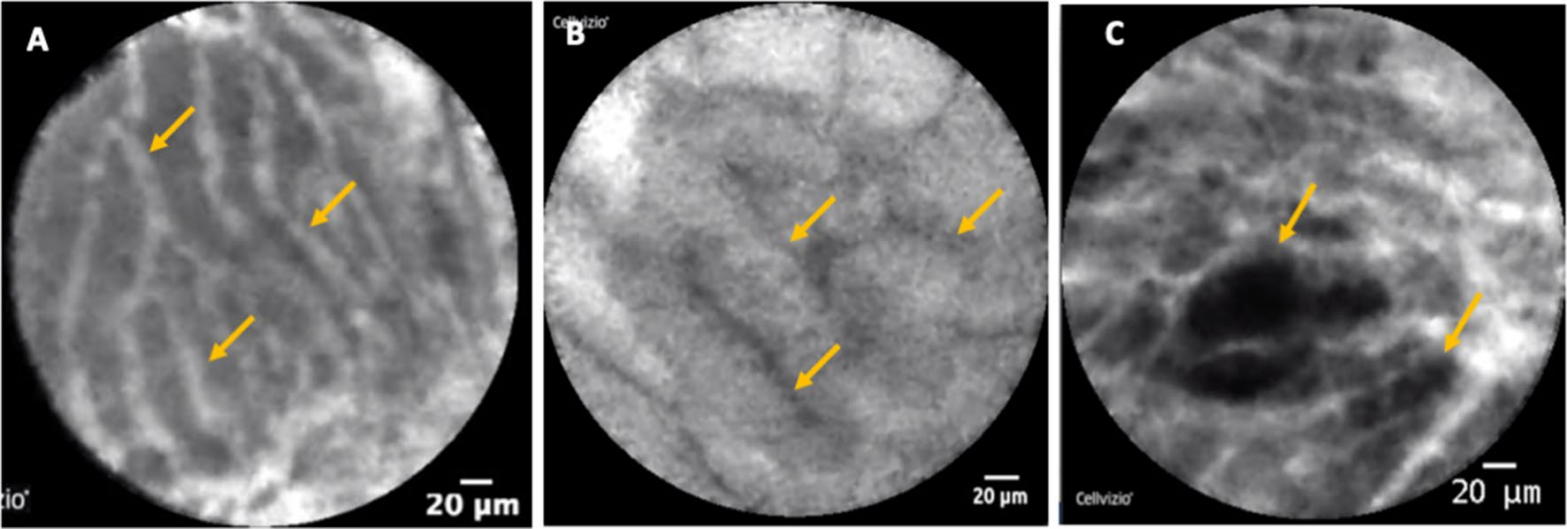
Common patterns of nCLE in pancreatic cystic tumors. **A** Serous cystadenoma: superficial vascular network in white (arrow) filled with fluorescein; black particles inside the vessels correspond to red cells. **B** IPMN: multiple papillae (arrows) with epithelial border in dark gray. **C** cystic *NEN* irregular clusters of tumoral cells (arrows)

The five independent pancreatic experts were informed about nCLE diagnostic performance by two nCLE experts (B.N and M.P) through teleconference. A slide deck was delivered summarizing the comprehensive diagnostic performance of nCLE criteria previously published for the most frequent types of PCLs (NEN, BD-IPMN, MCN, SCA, and PC) [18, 19]. Figure 1 provides illustrations of the common patterns of nCLE in PCLs. Prospectively validated nCLE diagnostic performances were presented, highlighting the very high specificity of nCLE for the diagnosis of SCA, IPMN, MCN and for the differentiation of mucinous from non-mucinous lesions [18, 19]. The five experts were also informed about nCLE limitations: first, the nCLE criterion “field of bright, gray or black particle” lacked specificity for PC characterization. It can be observed in other types of cysts such as the inflammatory cells that can be found in the cystic fluid of cystic tumors following infection, bleeding or previous procedures involving punctures; second, the nCLE criterion “dark spots of cell aggregates surrounded by gray areas of fibrosis and vessels” lacks specificity for NENs. It can be observed in other types of premalignant PCLs; third, the MCN nCLE criterion “epithelial borders” and the BD-IPMN nCLE criterion “papillae”, can be both observed in the same cyst, leading to the nCLE diagnosis of indeterminate mucinous lesions.

### Expert evaluations (Fig. 2)

The panel of experts independently reviewed every case blinded to one another’s decisions, to the final diagnoses and to patient therapeutic managements. Two evaluations were performed. The information for each patient was sequentially disclosed in a stepwise manner: progressively from clinical data, to EUS report and to FNA reports comprising histocytological analysis, CEA and amylase levels. nCLE data were not available in evaluation 1, referred to as “EUS-FNA” (the current standard of care for diagnosis). In evaluation 2, referred as “EUS-nCLE-FNA”, the nCLE report, describing prospectively observed nCLE criteria, their associated diagnoses and the quality of nCLE images, was given before the FNA reports. Patients were randomly reordered between evaluations 1 and 2. Evaluation 2 was performed at least 15 days after evaluation 1. For each patient and each evaluation, each expert had to retain one of the eight diagnosis and one of the three therapeutic management options.

**Fig. 2:**
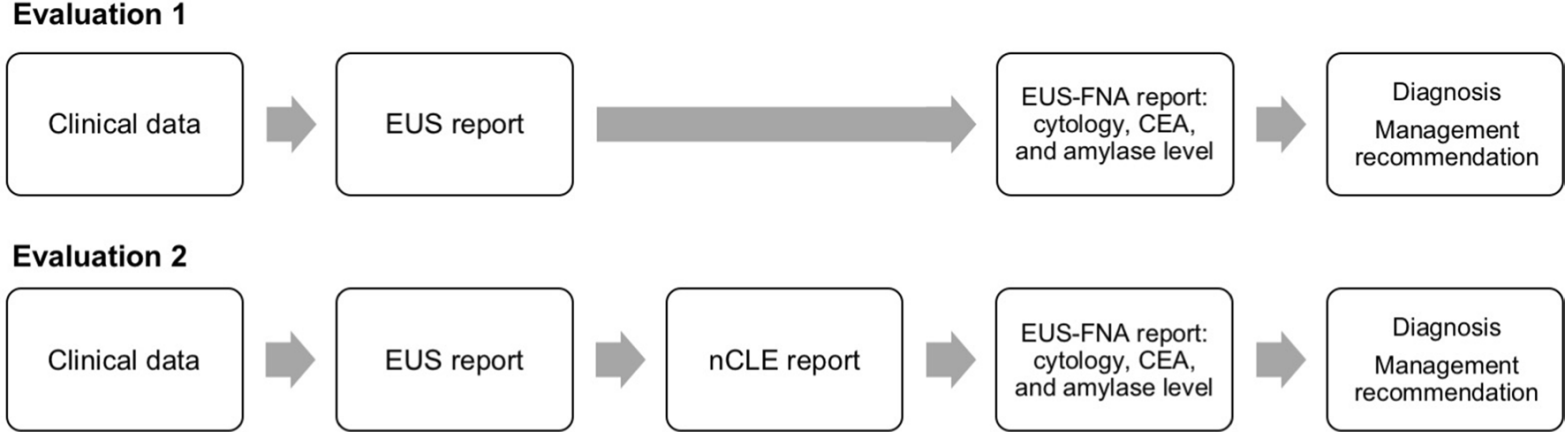
Flowcharts of evaluation 1 (EUS-FNA) and evaluation 2 (EUS-nCLE-FNA). *EUS-FNA* endoscopic ultrasound-guided fine-needle aspiration, *nCLE* needle-based confocal laser endomicroscopy, *CEA* carcinoembryogenic antigen

### Statistical analyses

Baseline characteristics, including demographic and clinical data, were described as percentages and ranges or means and standard deviations, as appropriate. Diagnostic yields were defined as the ratio of conclusive tests (nCLE and FNA cytohistology) over the total number of patients.

To assess interobserver agreement (IOA) between the five experts, Fleiss’ kappas were calculated for the proposed diagnoses and therapeutic management for evaluations 1 and 2. The R package “raters” was used to determine Fleiss’ kappa with 95% CI. Fleiss’ Kappa was interpreted using Landis and Koch-Kappa’s benchmark scale.

For each patient, final consensus on diagnosis and therapeutic management were determined with a majority of at least 3/5 of the experts. The absence of a final consensus led to indeterminate answers. A consensus on the diagnosis of indeterminate mucinous lesions was determined when a majority of experts proposed a diagnosis belonging to the overall group of mucinous lesions (IPMN, MCN, indeterminate mucinous lesion) in the absence of a majority for a more specific diagnosis (IPMN, MCN). The impact on diagnosis was defined as the change in the final consensus diagnosis between evaluations 1 and 2. The impact on therapeutic management was defined as the change in the final therapeutic management consensus between evaluations 1 and 2.

Overall diagnostic and therapeutic management proportion changes between evaluations 1 and 2 were compared using the *χ*^2^ square test. Changes between evaluations 1 and 2 regarding the specific diagnostic and therapeutic management categories were compared using McNemar’s test. Fleiss’ kappas from evaluations 1 and 2 were compared using the *Z* test. *p* < 0.05 was considered statistically significant.

### Endpoints

The main objective of the study was to evaluate the impact of nCLE on PCL therapeutic management: a significant proportion of final consensus changes between evaluations 1 and 2 for a given therapeutic management option was defined as the primary endpoint. The secondary objectives of the study were to evaluate the impact of nCLE on diagnosis and on IOAs for both diagnosis and therapeutic management: a significant proportion of final consensus changes for a given type of diagnosis, a significant increase of IOAs and an increased rate of full agreement between the five experts, between evaluations 1 and 2, for both diagnosis and therapeutic management, were also defined as secondary endpoints.

## Results

### Demographics and clinical data

A prospective database of patients with large single PCL examined by EUS-FNA and nCLE was derived from the CONTACT2 study (Supplementary Fig. 1). In this trial, 217 patients with PCLs were potentially eligible. Eight were excluded, leaving 209 eligible patients. With two failures of the puncture procedure and 1 nCLE imaging failure, EUS-nCLE-FNA procedures were successfully performed in 99% of the cases (206/209). The demographic and clinical data of the 206 analyzable patients are described in Tables 1 and 2. Overall FNA cytohistology was contributory in 30% of the cases (61/206). A conclusive nCLE test (identified nCLE criteria) was obtained in 175 patients, leading to an overall nCLE diagnostic yield of 85% (175/206). CEA was available in 76% of the cases (157/206), with a level > 192 ng/mL in 35% (55/157) of the cases and < 5 ng/mL in 38% (59/157) of the cases. Three cases of post-procedure pancreatitis (1.4%) were reported. No adverse events associated with fluorescein injection were noted.

**Table 1:** Demographic and clinical data for all cysts

Patients (*n* = 206)	
Age, mean (range), years	57 (23–84)
Male [*n* (%)]	69 (33)
Pre-existing conditions [*n* (%)]	
Previous EUS-FNA	64 (33)
Symptoms [*n* (%)]	
No symptoms	154 (75)
Acute pancreatitis	16 (8)
Aspecific abdominal pain	36 (17)
Cyst morphology	
Location [*n* (%)]	
Uncinate	10 (49)
Head	58 (28)
Neck	27 (13)
Body	67 (33)
Tail	44 (21)
Lesion size, mean (range), mm	38 (20–200)
Main pancreatic duct dilation [*n* (%)]	19 (9)
Number of cavities [*n* (%)]	
1	88 (43)
2	22 (11)
> 2 and < 10	58 (28)
≥ 10	38 (18)
Wall thickness ≥ 1 mm [*n* (%)]	32 (16)
Presence of calcification [*n* (%)]	18 (9)
Intracystic concentrations	
CEA > 192 ng/mL [*n* (%)]	55 (27)
CEA < 5 ng/mL [*n* (%)]	59 (29)
Amylase < 250 IU/L [*n* (%)]	63 (31)

*CEA* carcinoembryogenic antigen, *EUS*-*FNA* endoscopic ultrasound-guided fine-needle aspiration

**Table 2:** Technical feasibility and safety for all cysts

Cyst access [*n* (%)]	
Easy	188 (91)
Moderate or difficult	18 (9)
Needle type [*n* (%)]	
19 G Echo Tip Ultra*	21 (10)
19 G Expect Flexible^†^	185 (90)
Access route [*n* (%)]	
Transgastric	140 (68)
Transduodenal	66 (32)
Second part of the duodenum	5 (2)
Miniprobe extraction from the needle [*n* (%)]	
Possible	195 (95)
Not possible (extracted together with the needle)	11 (5)
Cytohistology [*n* (%)]	
Contributive	61 (30)
Non-contributive	145 (70)
Biochemical dosage [*n* (%)]	
Successful	157 (76)
Unsuccessful	49 (24)
*Insufficient fluid volume*	34 (17)
*Intracystic bleeding (hematic fluid)*	8 (4)
*High viscosity*	4 (1.9)
*Fluid leak*	3 (1.4)
Safety [*n* (%)]	
Post-procedure pancreatitis	3 (1.4)

*nCLE* needle-based confocal laser endomicroscopy

*Cook Medical Inc., Bloomington, Indiana, USA

^†^Boston Scientific Corp., Marlborough, Massachusetts, USA

### Impact of nCLE on PCL diagnoses

**Fig. 3:**
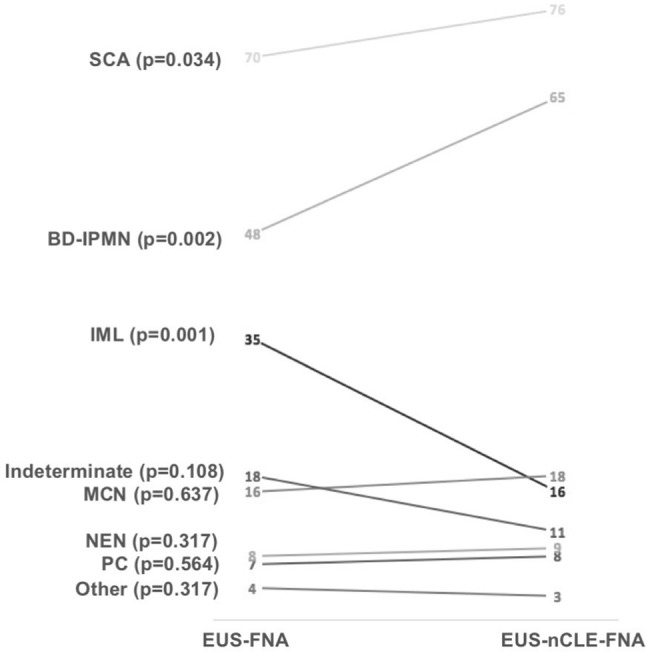
Diagnoses consensus in evaluations 1 (EUS-FNA) and 2 (EUS-nCLE-FNA). *BD-IPMN* branch duct-intraductal papillary mucinous neoplasm, *IML* indeterminate mucinous lesion, *MCN* mucinous cystadenoma, *SCA* serous cystadenoma, *PC* pseudocyst, *NEN* neuroendocrine neoplasm, *EUS* endoscopic ultrasonography, *FNA* fine-needle aspiration, *nCLE* needle-based confocal laser endomicroscopy. *p* values from McNemar test are indicated for each diagnostic option

The addition of nCLE information to standard EUS-FNA analysis significantly changed the proposed diagnoses in 27% of the cases (57/206) (*p* = 0.005) (Supplementary Table 1). Combined EUS-nCLE-FNA allowed a significant increase of BD-IPMNs diagnoses, from 48 to 65 (*p* = 0.002), significantly decreasing IML diagnoses from 35 to 16 (*p* = 0.001) (Fig. 3). Indeed, EUS-nCLE-FNA refined 23 indeterminate mucinous lesions into 16 BD-IPMNs and 7 MCNs (Supplementary Table S1). The number of SCA diagnoses also significantly increased from 70 to 76 (*p* = 0.034) (Supplementary Table S1). The number of indeterminate cysts numerically but not significantly decreased from 18 to 11 (*p* = 0.108) (Fig. 3). The addition of nCLE did not significantly impact the overall numbers of proposed NEN, MCN, PC, and other diagnoses (Fig. 3).

### Impact of nCLE on therapeutic management of patients with PCLs

**Fig. 4:**
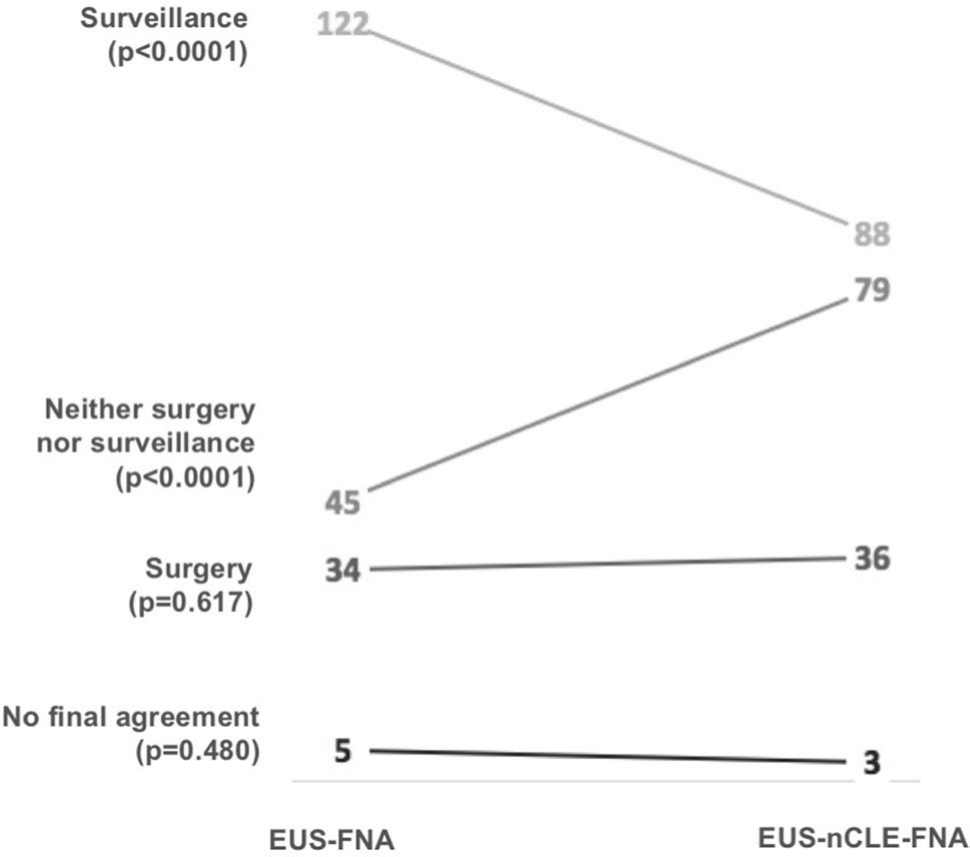
Therapeutic management according to evaluations 1 (EUS-FNA) and 2 EUS-nCLE-FNA). *EUS* endoscopic ultrasonography, *FNA* fine-needle aspiration, *nCLE* needle-based confocal laser endomicroscopy. *p* values from McNemar test are indicated for each diagnostic option

The addition of nCLE to the standard EUS-FNA procedure significantly changed 28% (58/206) of the overall proposed therapeutic managements. nCLE significantly increased the number of “neither surgery nor surveillance” recommendations, from 45 to 79 (*p* < 0.0001) and decreased surveillance recommendations, from 122 to 88 (*p* < 0.0001) (Fig. 4). The addition of nCLE to EUS-FNA analysis changed the therapeutic management for 33 patients from surveillance to “neither surgery nor surveillance” (Supplementary Table S2). Thirty-two of those 33 patients were diagnosed with SCAs by EUS-nCLE-FNA. The overall number of proposed surgeries was not statistically impacted by nCLE (Fig. 4). However, 6 of the 34 patients (18%) who were recommended for surgery in evaluation 1 were reconsidered for surveillance after the addition of nCLE information in evaluation 2, while 9 of 122 patients (7%) in the surveillance group in evaluation 1 were recommended for surgery in evaluation 2 (Supplementary Table 2). Those nine patients were diagnosed with mucinous lesions by EUS-nCLE-FNA.

### Impact of nCLE on interobserver agreements

**Fig. 5:**
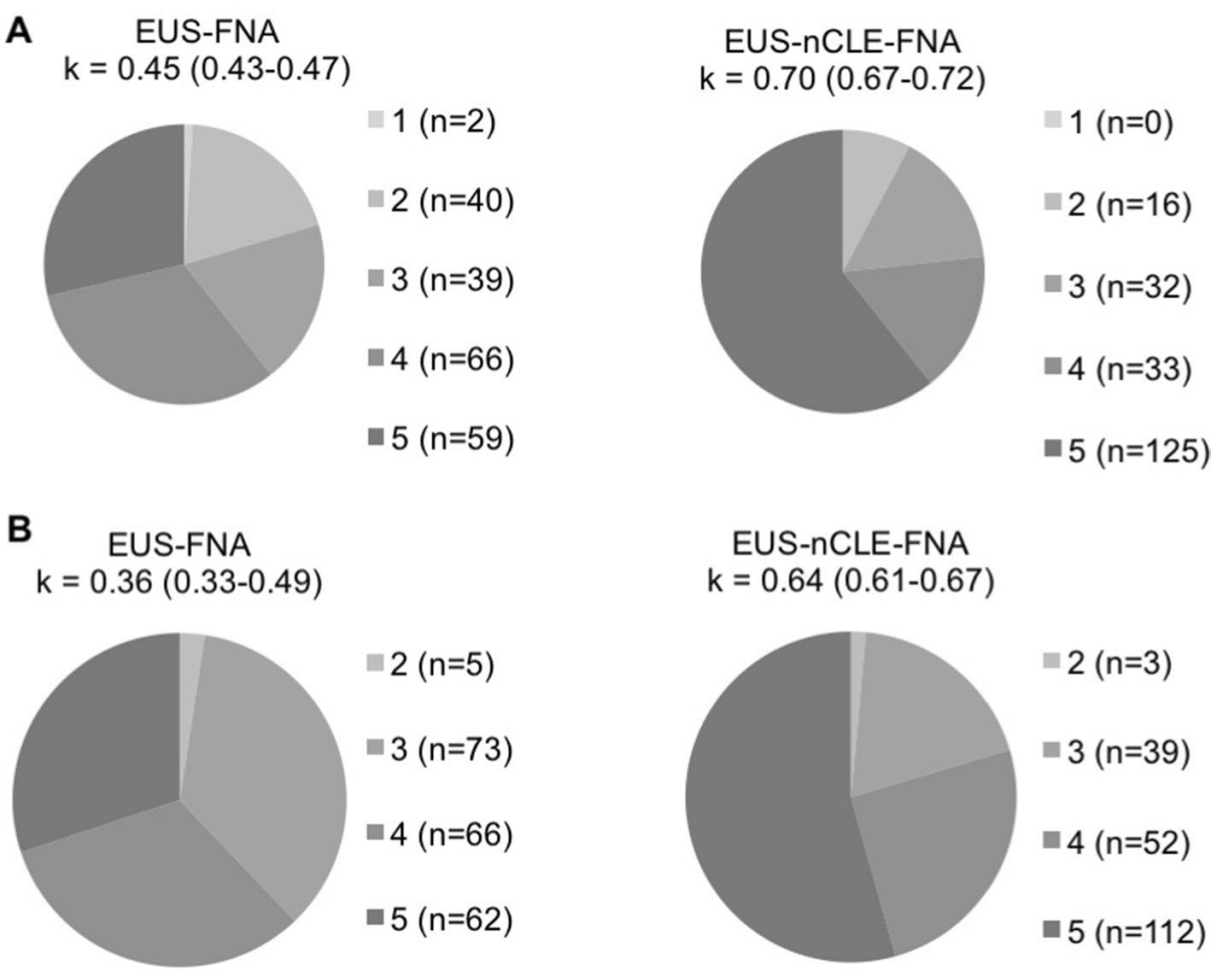
Agreement between the panel of five experts. **A** Agreement among the eight possible diagnosis options (NEN, BD-IPMN, MCN, indeterminate mucinous lesion, SCA, PC, indeterminate or other) according to evaluation 1 (EUS-FNA) and evaluation 2 (EUS-nCLE-FNA). **B** Agreement among the three possible therapeutic management options (“neither surgery nor surveillance”, “surveillance” or “surgery”) according to evaluation 1 and evaluation 2. *EUS* endoscopic ultrasonography, *FNA* fine-needle aspiration, *nCLE* needle-based confocal laser endomicroscopy, *k* Fleiss’ kappa. *NEN* neuroendocrine neoplasm, *BD-IPMN* branch duct-intraductal papillary mucinous neoplasm, *MCN* mucinous cystadenoma, *SCA* serous cystadenoma, *PC* pseudocyst

Regarding proposed diagnoses, EUS-nCLE-FNA Fleiss’ kappa was substantial (0.70, CI 95% 0.67–0.72) in the overall population of cysts and was significantly increased by 0.25 compared to that of EUS-FNA (0.45, CI 95% 0.43–0.47) (*p* < 0.0001). nCLE increased the rate of full agreement on diagnosis among the five experts from 29 (59/206) to 61% (125/206) (*p* < 0.0001) (Fig. 5A). For proposed therapeutic management, EUS-nCLE-FNA Fleiss’ kappa was substantial (0.64, CI 95% 0.61–0.67) in the overall population of cysts and was significantly increased by 0.28 compared to that of EUS-FNA (0.36, CI 95% 0.33–0.49) (*p* < 0.0001). nCLE increased the rate of full agreement on therapeutic management among the five experts from 30% (62/206) to 54% (112/206) (*p* < 0.0001) (Fig. 5B).

All authors had access to the study data and reviewed and approved the final version of the manuscript.

## Discussion

Increasing numbers of incidental PCLs have been identified due to increasing use of computed tomography (CT) scans and magnetic resonance imaging (MRI) [20, 21]. Single non-communicating PCL without chronic calcified pancreatitis represents the most challenging diagnostic issue and should be carefully considered because of the malignant potential of some types of PCLs. In current practice, the therapeutic management of PCLs usually relies on multidisciplinary review board decisions based on cyst imaging characteristics, CEA levels and cytohistopathological analyses. Using this approach for 206 patients extracted from a prospective database, the IOA Fleiss’ kappas between a panel of experts in pancreatic diseases were dramatically low for both PCL diagnosis (0.45) and therapeutic management (0.36). These results can be explained by the low rate of 30% of contributory FNA cytohistopathological analyses and by the lack of accuracy of CEA level. These data are consistent with those of the literature [22] and emphasize the necessity of a more reliable diagnostic method for the diagnosis of PCLs.

In our series, the overall diagnostic yield of nCLE (85%) was significantly higher than that of FNA cytohistopathology (30%). The addition of nCLE to EUS-FNA allowed significant changes of diagnoses and therapeutic managements in 27% and 28% of the cases, respectively (with *p* = 0.005 and *p* < 0.0001, respectively), while IOA Fleiss’ kappas among the experts significantly increased from 0.45 to 0.70 for diagnosis and from 0.36 to 0.64 for therapeutic management strategies. Strikingly, nCLE increased the rate of full agreement among the panel of experts from 29 to 61% for diagnoses and from 30 to 54% for therapeutic managements. This effect of nCLE increased the value of multidisciplinary review boards with less disagreement among participants.

The addition of nCLE significantly increased the number of BD-IPMNs diagnoses (*p* = 0.002) at the expense of a significant decrease of IML (*p* = 0.001). Indeed, EUS-nCLE-FNA refined 23 indeterminate mucinous lesions into 16 BD-IPMNs and 7 MCNs. These data emphasize that nCLE is useful to ascertain the diagnosis of BD-IPMN and should be recommended for a more specific diagnosis. nCLE also increased the number of SCA diagnoses (*p* = 0.034) and the number of “neither surgery nor surveillance” recommendations (*p* < 0.0001) while decreasing surveillance recommendations (*p* < 0.0001); the surveillance rate of SCAs fell from 40 (28/70) to 5% (4/76). These data show that nCLE is essential to ascertain the diagnosis and the therapeutic management of SCA by allowing the identification of its specific “superficial vascular network”. nCLE should be at least recommended to ascertain the presumptive diagnosis of SCA. Moreover, a recent French health economic model reported that nCLE may be cost-effective for the therapeutic management of SCA [23]. In this medico-economic analysis, the additional cost per patient for nCLE was estimated at 600€. This analysis showed that the addition of nCLE to EUS-FNA resulted in a reduction of 23% in the total rate of surgical intervention, which translated to a reduction in clinical costs of 13% (public sector) and 14% (private sector). Therefore, nCLE should be systematically considered when EUS-FNA is indicated for the evaluation of PCLs.”

Our data confirmed the safety of nCLE in the largest multicentric cohort of 206 patients who underwent EUS-FNA+nCLE procedures. Indeed, the rate of post-procedure acute pancreatitis in the trial was 1.4% (3/206). This incidence is similar to the previously reported risk levels following standard EUS-FNA procedures [24] and is lower than those of nCLE pilot studies [12]. The necessity of using a 19G needle to perform nCLE procedure represents the main technical limitation. Two technical failures of cyst puncture were observed in lesions only accessible from the second part of the duodenum, which is consistent with previously reported experience [15].

Our study had several limitations: (1) only a few of the PCLs included in the study had a surgical gold standard-based diagnosis. This is inherent to the therapeutic management of the studied disease, especially when dealing with presumptive benign PCLs for which ethical considerations obviously prevent systematic resections. For the other PCLs, the follow-up was not very long term. Therefore, the diagnoses proposed by the experts are tentative. Thus, the only certain finding is that the use of nCLE increases interobserver agreement between specialists. The comparison of retrospective data analyses with a prospectively obtained gold standard diagnosis would have introduced a major methodological bias. Nevertheless, two large prospective studies have already validated the very high diagnostic performance of nCLE for all types of cysts [17, 18] and have demonstrated the almost perfect interobserver reliability of nCLE criteria [17]. (2) The contribution of CT and MRI outcomes was not evaluated in our study due to the heterogeneity of data collection. Nevertheless, this would probably not have affected the results because the patients included in our cohort were sent for EUS-FNA after inconclusive CT and MRI. (3) We did not include in our study new PCLs diagnostic tools for which the potential interests have been published during the conduct of the study, including DNA mutations [25, 26], string sign tests [27], and EUS-guided microforceps biopsies [28]. (4) For the sake of simplification, therapeutic management was based on a consensus of a panel of five experts. Systematic resection was recommended for all patients diagnosed with MCN, whereas surveillance was recommended for BD-IPMN without worrisome features [1]. This can be discussed as the most recent guidelines propose observation as an option for asymptomatic MCNs < 4 cm without mural nodules [22, 29]. Nevertheless, MCNs are predominantly present in the body or tail of the pancreas of middle-age women and are definitely cured without the need for follow-up in the absence of cancer. The natural history of MCN remains unknown and non-operative management would require years of follow-up based on high-resolution imaging associated with high costs [1]. Therefore, in most centers, resection remains proposed for MCN.

## Conclusion

The diagnosis of large single pancreatic cysts represents a major issue for clinicians. The addition of nCLE information to EUS-FNA significantly improves the *reliability both in diagnosis and therapeutic management among experts* in pancreatic cystic lesions. These results support the recognition of nCLE as a key tool of the standard of care for such clinical situations.

## Electronic supplementary material

Below is the link to the electronic supplementary material.

Supplementary material 1 (DOCX 152 kb)

Supplementary material 2 (DOCX 67 kb)

## Abbreviations

BD-IPMN: Branch duct-intraductal papillary mucinous neoplasm
CT: Computed tomography
CEA: Carcinoembryonic antigen
EUS-FNA: Endoscopic ultrasound fine-needle aspiration
IML: Indeterminate mucinous lesion
MCN: Mucinous cystic neoplasm
MRI: Magnetic resonance imaging
nCLE: Needle-based confocal laser endomicroscopy
NEN: Neuroendocrine neoplasm
PC: Pseudocyst
PCLs: Pancreatic cystic lesions
SCA: Serous cystadenoma
