# Supplementary material for: Impact of needle-based confocal laser endomicroscopy on the therapeutic management of single pancreatic cystic lesions
Source: Surg Endosc. 2019 Aug 13;34(6):2532–40. doi: 10.1007/s00464-019-07062-9 (PMC7214514; doi:10.1007/s00464-019-07062-9)
Supplement: Supplementary file 1 — Supplementary material 1 (DOCX 152 kb) [file 464_2019_7062_MOESM1_ESM.docx]

**Supplementary Figure Legend**

**Supplementary Figure 1: Patient Study Flow Chart.** PCL, pancreatic cystic lesions; EUS-FNA, endoscopic ultrasound-guided fine needle aspiration**;** nCLE, needle-based confocal laser endomicroscopy

**
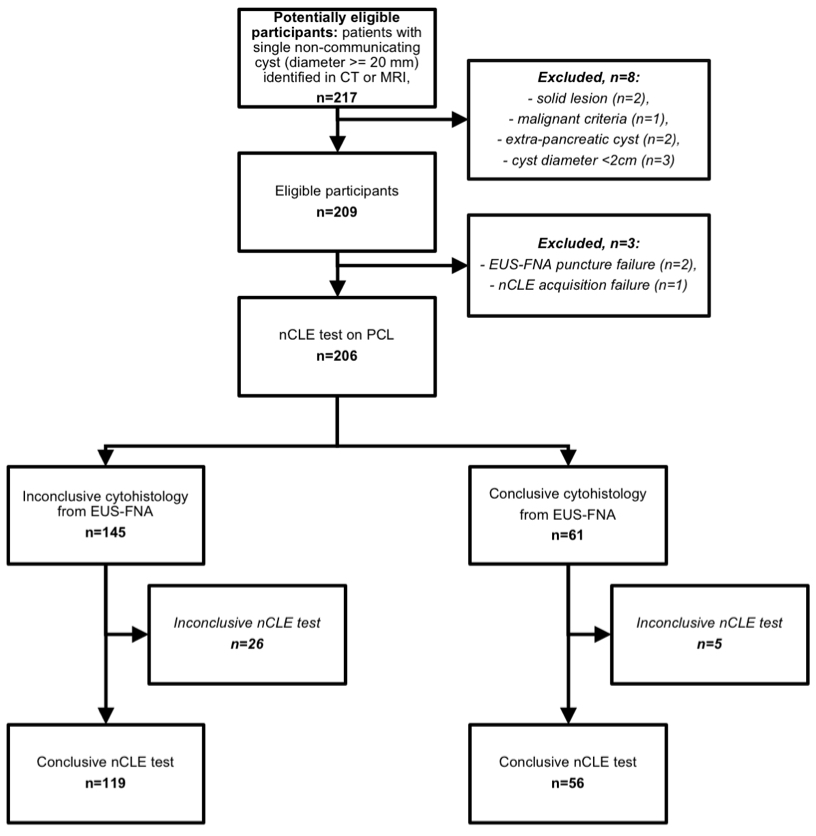
**
