# Supplementary material for: Impact of needle-based confocal laser endomicroscopy on the therapeutic management of single pancreatic cystic lesions
Source: Surg Endosc. 2019 Aug 13;34(6):2532–40. doi: 10.1007/s00464-019-07062-9 (PMC7214514; doi:10.1007/s00464-019-07062-9)
Supplement: Supplementary file 2 — Supplementary material 2 (DOCX 67 kb) [file 464_2019_7062_MOESM2_ESM.docx]

|  | **EUS-nCLE-FNA** | | | | | | | | |
| --- | --- | --- | --- | --- | --- | --- | --- | --- | --- |
| **EUS-FNA** | **No Change** | **NEN** | **BD-IPMN** | **MCA** | **IML** | **SCA** | **PC** | **Other** | **Indeterminate** |
|  | **NEN** | 7 |  |  |  |  |  |  |  |
|  | **BD-IPMN** |  | 42 | 1 | 3 |  |  |  | 2 |
|  | **MCA** |  | 2 | 8 | 5 |  | 1 |  |  |
|  | **IML** |  | 16 | 7 | 8 | 1 | 1 |  | 2 |
|  | **SCA** |  | 1 |  |  | 69 |  |  |  |
|  | **PC** |  |  |  |  |  | 7 |  | 1 |
|  | **Other** |  |  |  |  |  |  | 3 | 1 |
|  | **Indeterminate** | 1 | 4 | 2 |  | 6 |  |  | 5 |

**Supplementary Table 1: Impact of nCLE on the diagnoses of pancreatic cystic lesions**. Contingency table depicting the 57 diagnostic changes between paired EUS-FNA and EUS-nCLE-FNA outcomes. NEN, neuroendocrine neoplasm; BD- IPMN, branch-duct intraductal papillary mucinous neoplasm; MCA, mucinous cystadenoma; IML, indeterminate mucinous lesion; SCA, serous cystadenoma; PC, pseudocyst. EUS-FNA, endoscopic ultrasound-guided fine needle aspiration; nCLE, needle-based confocal laser endomicroscopy.

|  | **EUS-nCLE-FNA** | | | | |
| --- | --- | --- | --- | --- | --- |
| **EUS-FNA** | **No Change** | **Surgery** | **Surveillance** | **No Surveillance**  **No surgery** | **No Final Agreement** |
|  | **Surgery** | 27 | 6 |  | 1 |
|  | **Surveillance** | 9 | 78 | 33 | 2 |
|  | **No surveillance**  **No surgery** |  | 2 | 43 |  |
|  | **No final agreement** |  | 2 | 3 |  |

**Supplementary Table 2: Impact of nCLE on the therapeutic management of patient with pancreatic cystic lesions.** Contingency table depicting the 58 therapeutic management changes per patient between paired EUS-FNA and EUS-nCLE-FNA outcomes. EUS-FNA, endoscopic ultrasound-guided fine needle aspiration; nCLE, needle-based confocal laser endomicroscopy.
